# Electronic correlations in epitaxial CrN thin film

**DOI:** 10.1038/s41598-023-42733-7

**Published:** 2023-09-25

**Authors:** Shailesh Kalal, Sanjay Nayak, Sophia Sahoo, Rajeev Joshi, Ram Janay Choudhary, Rajeev Rawat, Mukul Gupta

**Affiliations:** 1 UGC-DAE Consortium for Scientific Research, University Campus, Khandwa Road, Indore, 452 001 India; 2 Department of Physics, Chemistry and Biology (IFM), Linköping University, 581 83 Linköping, Sweden

**Keywords:** Condensed-matter physics, Applied physics

## Abstract

Chromium nitride (CrN) spurred enormous interest due to its coupled magnetostructural and unique metal-insulator transition. The underneath electronic structure of CrN remains elusive. Herein, the electronic structure of epitaxial CrN thin film has been explored by employing resonant photoemission spectroscopy (RPES) and X-ray absorption near edge spectroscopy study in combination with the first-principles calculations. The RPES study indicates the presence of a charge-transfer screened 3$d ^n{\underline{L}}$ ($L$: hole in the N-2$p$) and 3$d ^{n-1}$ final-states in the valence band regime. The combined experimental electronic structure along with the orbital resolved electronic density of states from the first-principles calculations reveals the presence of Cr(3$d$)-N(2$p$) hybridized (3$d ^n{\underline{L}}$) states between lower Hubbard (3$d ^{n-1}$) and upper Hubbard (3$d ^{n+1}$) bands with onsite Coulomb repulsion energy (U) and charge-transfer energy ($\Delta$) estimated as $\approx$ 4.5 and 3.6 eV, respectively. It verifies the participation of ligand (N-2$p$) states in low energy charge fluctuations and provides concrete evidence for the charge-transfer ($\Delta<$U) insulating nature of CrN thin film.

## Introduction

Transition metal nitride (TMN) materials have drawn considerable attention because of their interesting physical properties: mechanical strength, ultra-hardness, corrosion resistance, high-melting points, superconductivity, thermoelectricity, magnetostructural phase-transition^1–5^ etc. These properties are primarily governed by the electronic structure^1,6,7^. Electronic correlations play an indispensable role in determining exotic properties of strongly correlated materials such as high-temperature superconductivity^8^ and multiferroicity^9^. Mott metal-insulator transition (MIT) is one such phenomenal example. The MIT depends on the competition between itinerancy and electron–electron correlations^10,11^. The Mott–Hubbard theory^12^ first described the MIT via a reduction of the W/U parameter, where W is the bandwidth and the Hubbard energy U is the effective onsite Coulomb repulsion. Afterwards, Zaanen et al.^13^ proposed a classification scheme for TM based compounds. According to this scheme, depending on the magnitude of ligand to metal charge-transfer energy ($\Delta$) or intra-atomic Coulomb repulsion energy (U), the TM compounds fall into two categories: (i) Mott–Hubbard (U$<\Delta$) (ii) charge transfer ($\Delta<$U) insulator.

Among TMNs, CrN indeed seems to be peculiar as it does not shows superconductivity like its isostructural neighbors: TiN^14^, VN^15^, NbN^16^, MoN^17^ etc. It shows a first-order magnetostructural transition from a high-temperature paramagnetic cubic (Fm-3m) to a low-temperature antiferromagnetic (AFM) orthorhombic (Pnma) phase. In the literature, the transition temperature (T$_N$) of CrN varies from nearly room temperature to 100 K or even gets completely suppressed depending on the Cr/N ratio^18^, polycrystalline/epitaxial nature, compressive/tensile strain^19,20^, thickness^19,21^, choice of substrates  as well as substrate orientations^20,22^. Given the fundamental^2,23,24^ and technological interest^25^, some experimental and theoretical works have already been done on CrN and the structural and magnetic properties of CrN are well understood. However, the optical and electrical properties of CrN have been rather controversial and the underlying electronic structure of CrN received significant attention^19,20,22,23,26–30^. Quintela et al.^29^ found semiconducting behavior in the paramagnetic phase with an activation energy of 75 meV but concluded that in the AFM phase the electrical resistivity behavior was neither conventional semiconducting nor fully itinerant. Herle et al.^31^ showed bulk CrN follows activation behavior with a small gap of 90 meV in the temperature range of 5–300 K. Constantin et al.^32^ reported a high temperature semiconductor (band gap $\approx$ 50 meV) to a low temperature metallic transition around 240 K in epitaxial CrN thin film deposited on MgO (001) substrate. Further, Bhobe et al.^23^ reported similar results and concluded that the bulk CrN exhibits a high temperature correlated insulator (band-gap $\approx$ 70 meV) to a disordered metal transition. In a recent work by Jin et al.^19^ demonstrated that strain mediated orbital splitting can customize the small band-gap $\approx$ 20 meV which drives MIT in the epitaxial thin films or free standing foil of CrN.

Besides this, Imada et al.^11^ interpreted the observed magnetic, structural, and electronic properties of CrN in terms of charge ordering or Mott-insulating behavior, which is characteristic of correlated electron systems. Similarly, Herwadkar et al.^26^ performed first-principles calculations using the local spin-density approximation corrected by the Hubbard Coulomb term (LSDA + U) and showed a small spin separation of states near the Fermi-level (E$_F$) which open a small charge-gap of less than 1 eV between the N-2$p$ and Cr-3$d$ bands hinting that the CrN is a charge-transfer type insulator. Moreover, Allah et al.^33^ studied the electronic and vibrational properties of polycrystalline CrN using optical transmission and reflection measurements. They reported different absorption bands in the frequency range of 0.012–2.48 eV and explained these bands in terms of the charge-transfer insulator picture. The similar optical reflectance measurements in broad frequency ranges (0.04–5 eV) have been performed by Gall et al.^34^. They showed a small indirect-band gap of 0.19 eV at the E$_F$, which was attributed to electron interaction effects and claimed CrN to be a Mott–Hubbard-type insulator. Hence, a comprehensive knowledge of the electronic structure comprising the experimental and theoretical electronic band structure is lacking in CrN. To solve the discrepancy in electronic state of the CrN, a detailed study underlying the electronic structure of CrN is required.

In view of contradictory experimental and theoretical reports on the electronic structure of CrN, the present study attempts to investigate the electronic structure of CrN. To explore the true electronic structure and eliminate any strain-related modifications^19,30^ relaxed thin film of CrN has been deposited on MgO (001) substrate. We have investigated occupied and unoccupied density of states using a combination of resonant photoemission spectroscopy (RPES) and X-ray absorption near edge structure (XANES) measurements in combination with first-principles calculations. Finally, the detailed electronic structure in the vicinity of E$_F$ has been examined.

## Results and discussion

### Structural and transport properties

**Figure 1:**
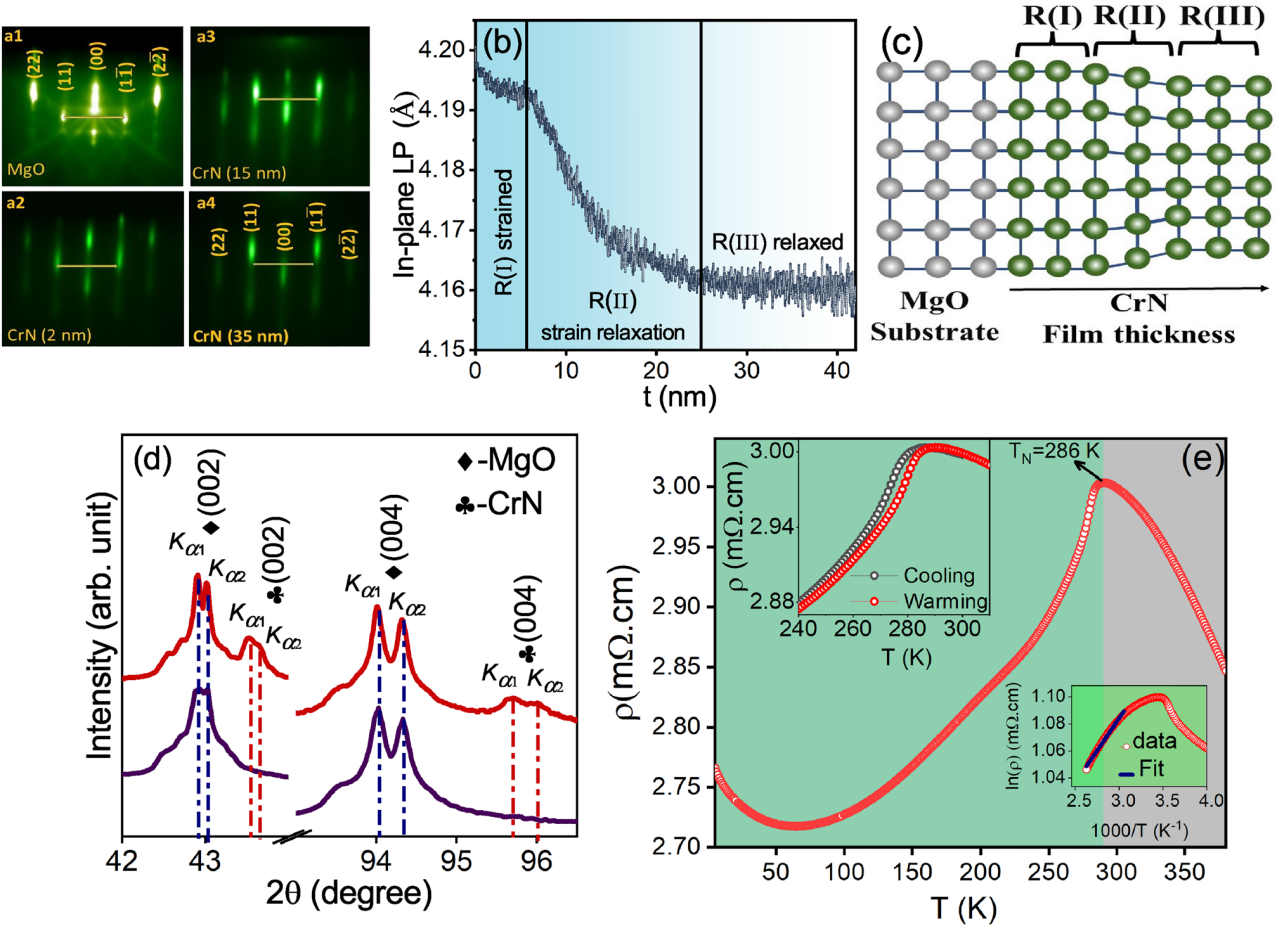
(**a1**–**a4**) shows the RHEED images taken along (110) direction of the MgO (001) substrate and deposited CrN thin film at thickness (t) of 2, 15 and 35 nm. (**b**) In-plane lattice parameter as a function of film thickness (t). (**c**) Schematic shows the strain relaxation process. (**d**) X-ray diffraction patterns of grown CrN thin film along with bare MgO (001) substrate. (**e**) Temperature dependent electrical resistivity measurements with upper inset shows expanded view of heating and cooling cycle and lower inset shows activation fit in the high temperature regime.

Figure 1a1 show RHEED patterns taken along (110) direction of bare MgO (001) substrate and (a2–a4) are the images taken during the film growth at 2, 12 , 35 nm. The in-plane lattice parameter (LP) was obtained by monitoring spacing between (11) and (1${\bar{1}}$) diffraction streaks (marked in the RHEED images) as a function of film thickness (t) (see Fig. 1b). Based on the strain relaxation this spectrum is apportioned into three regions R(I), R(II) and R(III): In R(I), at the early stages of growth (0–5 nm), grown CrN film is fully strained and the estimated in-plane LP $\approx$ 4.19 ± 0.01 Å  alike to MgO (LP of MgO $\approx$ 4.21 Å) substrate. The respective RHEED image taken after $\approx$ 2 nm deposition is shown in Fig. 1a2. Here, the intense streaky pattern suggests film follows the substrate orientation and grows in 2D layer-by-layer manner^35^. In R(II) t = 5 to 25 nm, as can be seen from Fig. 1b strain relaxation ensues and LP of growing film approaches the bulk values^19^. The RHEED image (taken at t $\approx$ 15 nm) is presented in Fig. 1a3 depicting a modulated streaky pattern evincing that the film has a multilevel stepped surface possibly due to enhancement in the surface roughness. Further, in R(III) t>25 nm: LP of the growing film becomes constant ($\approx$ 4.16 Å) which endows that the film is now relaxed^19^. The modulated streaky RHEED pattern shown in the Fig. 1a4 taken at t = 35 nm confirms the cube on cube symmetric growth of CrN thin film on MgO (001) substrate ended with multilevel stepped surface. Figure 1c shows a schematic depicting the grown film possess in-plane (out-of-plane) tensile (compressive) strain in the R(I) region and as the thickness of the film increases some strain relaxation takes place in R(II). Finally, in the R(III) film is fully relaxed. Figure 1d shows out-of-plane XRD patterns of CrN thin film taken after the film deposition and bare MgO (001) substrate. It shows single-phase growth of CrN thin film along (001) direction. The out-of-plane LP of the film is calculated to be 4.160 ± 0.005 Å. Though, N-vacancies in CrN are seems to be thermodynamically stable^36^, with a right choice of growth parameters, stoichiometric CrN films can be grown on MgO (001) substrate^37^. Zhang et al.^38^ showed that the deviation in the CrN composition distort the cubic symmetry and drastically changes the c/a ratio, resulting in an overall lattice shrinkage. While in our grown film both in-plane and out-of-plane LP values akin to the bulk values of $\approx$ 4.16 Å (c/a $\approx$ 1) which is an indication stoichiometric nature of the grown film.

The temperature-dependent resistivity measurement depicted in Fig. 1e, reveals an anomaly near the room temperature regime. Further, the inset of Fig. 1e demonstrates clear hysteresis in the cooling and warming cycle, confirming a first-order phase transition with a T$_N$ $\approx$ 286 K. This value matches well the bulk values and confirms the stoichiometric nature of the film^2,23^. The $\rho$ follows an activation behavior in the high temperature regime (see lower inset of Fig. 1e). Herein, $\rho$(T) has been fitted using the expression: $\rho$(T)=$\rho$(0)e$^{-E_g/2K_BT}$, a linear fit to ln($\rho$) vs 1/T yields a band gap of $\approx$ 26 meV confirms the opening of a finite gap. However, in the low-temperature regime (below 60 K), the resistivity ($\rho$) exhibits a negative temperature coefficient without following any activated behavior. This behavior has been widely reported in the literature^23,28,29^. In a correlated antiferromagnetic metal, itinerant electrons may become frozen or crystallized due to interactions with localized spins and with each other, leading to reduced mobility. Consequently, the resistivity increases as the temperature approaches absolute zero (T $\rightarrow$ 0)^39,40^. It should be noted that a comprehensive analysis of the magnetic properties of the film grown in this study will be reported elsewhere.

### Electronic properties

#### valence band spectra

**Figure 2:**
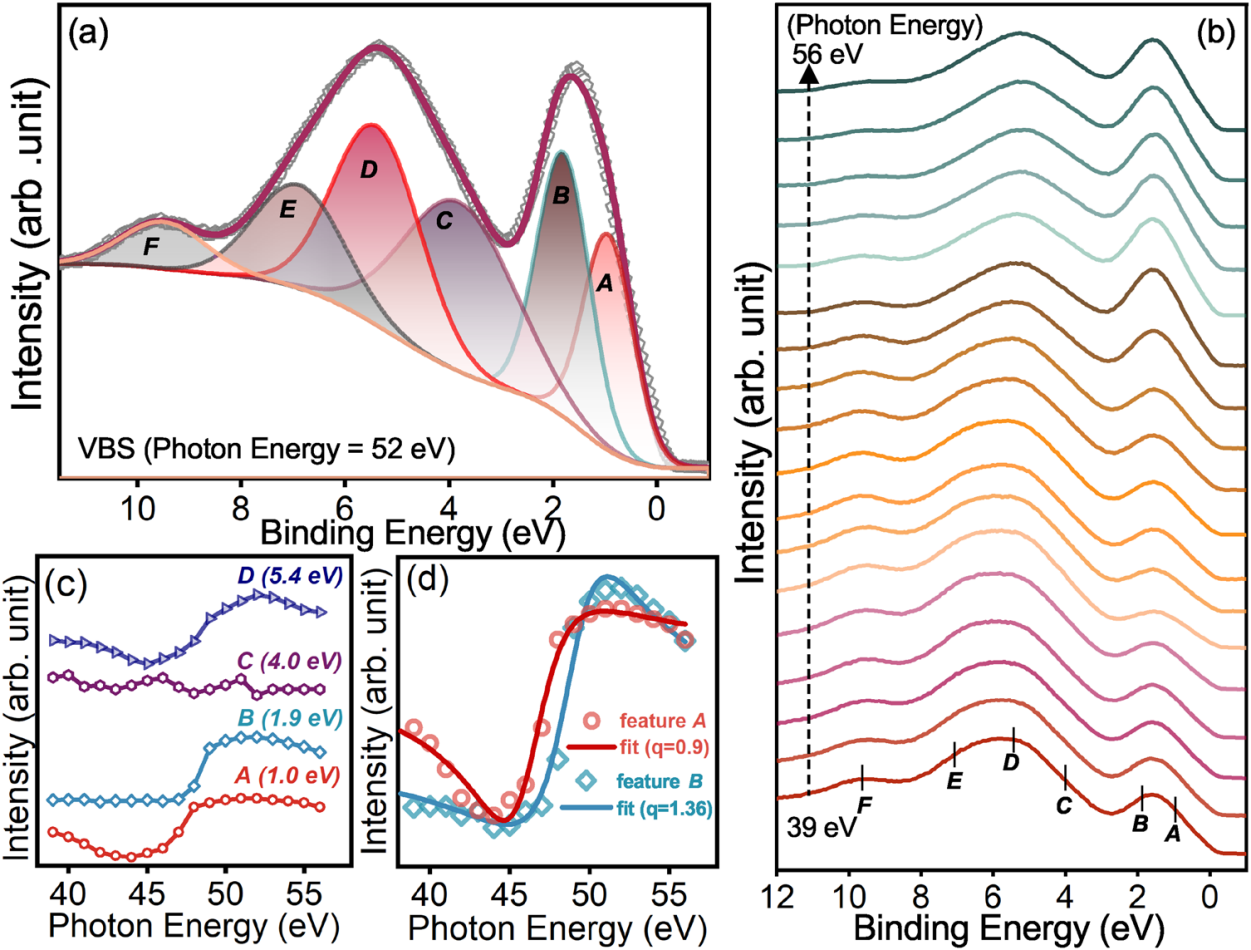
(**a**) Valence band spectrum of CrN thin film taken at 52 eV photon energy deconvoluted using peaks labeled *A* to *F*. (**b**) Energy distribution curves (EDCs) of the VBS obtained for photon energies between 39 and 56 eV. (**c**) Constant initial state (CIS) plot of *A*, *B*, *C*, *D* features in valence band of CrN thin film. (**d**) Fano-line shape fit of the CIS plot of feature *A* and *B*. The PES measurements have been carried out at 300 K.

Figure 2a shows the valence band spectrum (VBS) of CrN thin film recorded at the incident photon energy of 52 eV. The estimated band gap from electrical resistivity measurements is $\approx$ 26 meV. However, the finite DOS at E$_F$ in VBS is visible due to limited instrumental resolution ($\approx$ 300 meV). In the VBS, an intense peak around 2 eV binding energy (BE) and the broad feature has been observed between 3 and 10 eV. Also, an overall VB spectral shape concurrent with earlier reports^23,41^. The spectral features appearing near the E$_F$ are mostly dominated by the Cr-3$d$ derived states, while the broad feature at the higher BE has a significant N-2$p$ band contribution^23^. The electronic structure calculations for CrN using different exchange correlation potentials and hybrid functions^26,42,43^ showed presence of a considerable N-2$p$ character near E$_F$. Hence, to understand the contribution of different spectral bands, VBS is deconvoluted using *A*–*F* Voight peaks that adequately reproduces the major features of the spectrum (see Fig. 2a). To understand the origin of different features in VBS, resonant photoemission spectroscopy (RPES) measurement has been performed and discussed in the next section.

#### Resonant photoemission spectroscopy

In RPES, valence band spectra of the film were recorded by sweeping the photon energy across Cr-3$p \rightarrow$3$d$ excitation threshold. Figure 2b shows the energy distribution curves (EDCs) of film with the photon energy varying from 39 to 56 eV. Herein, the sharp resonance around 52 eV is due to the quantum-mechanical interference between two excitation processes, which transform a certain initial state to the same final state via two possible channels. One channel is due to the direct photoemission from Cr-3$d$ states^44^:

1 $$\begin{aligned} \mathrm {Cr: 3p^63d^3(t^3_{2g})+h}\upsilon \rightarrow \mathrm {Cr:3p^63d^2(t^2_{2g})+e^-} \end{aligned}$$

and the second channel of photoemission comes from the intra-atomic excitation process at the resonant photon energy 52 eV by the Cr-3p state followed by super Coster–Kronig decay, represented as:

2 $$\begin{aligned} \mathrm {Cr:3p^63d^3(t^3_{2g})+h}\upsilon \rightarrow \mathrm {[3p^53d^4]^*\rightarrow Cr:3p^63d^2(t^2_{2g})+e^-}. \end{aligned}$$

The final state is indistinguishable in the two cases thus the Cr-3$d$ photoelectron yield rapidly enhances and exhibits resonance.The variation of spectral intensity of different VBS features with respect to the incident photon energy is visualized using the constant initial state (CIS) plot, illustrated in Fig.2c by plotting the area under curve with respect to photon energy for different features. Here, CIS plot of the feature *B* exhibits only resonance with maxima around 52 eV while the features *A* and *D* show strong resonance enhancement with a considerable anti resonance dip. Feature $C$ at BE $\approx$ 4.0 eV does not show any resonance, confirming the non-bonding nature of the N-2$p$ band. Furthermore, feature $E$ and $F$ are identified as the satellite structure of Cr^44,45^.

It is noteworthy that the CIS spectra for the 3$d ^{n-1}$ final-states show only resonance peak without a remarkable anti-resonance dip near the TM 3$p$-3$d$ threshold, while for 3$d ^n{\underline{L}}$ (${\underline{L}}$ denotes a hole in the ligand-2$p$ band) final-states, an anti-resonance dip on the lower photon energy side of a shallow peak is accentuated. Thus, the presence of anti-resonance dip followed by the sharp resonance in the CIS plot of feature *A* (1.0 eV) and *D* (5.4 eV) reveals these bands have a strong hybridized Cr 3$d$-N 2$p$ (3$d ^3{\underline{L}}$ final-state) band character. For better visualization, the CIS plot of features *A* and *B* are fitted using a Fano line shape (see Fig. 2d) Eq. (3)^46^ given by:

3 $$\begin{aligned} \mathrm {I(hv)=I_0\frac{(q+E)^2}{1+E^2}+I_{NR}(hv)}, \end{aligned}$$

where I$_0$(hv) is the 3$d$ emission in the absence of the autoionizing transition, $\mathrm {I_{NR}(hv)}$ is the non-interfering background contribution, $\mathrm {E=(hv-E_R)/\varGamma }$: $\mathrm {E_R}$ and $\varGamma$ are energy and the width of the transition and q is the asymmetry parameter determined by the magnitude and sign of the transition and interaction matrix elements. In general, q is lower for hybridized ligand 2$p$ and transition metal 3$d$ states and higher for pure TM 3$d$ states^47,48^. In case of CrN thin film, feature *A* shows a dip in the CIS spectra and fitted well using lower q value (= 0.9) indicating their strong hybridized N-2$p$ and Cr-3$d$ (3$d ^3{\underline{L}}$ final-state) band character while the higher value of q (1.36) for feature *B* confirming its pure Cr-3$d$ (3$d ^2$ final state) band character. To the best of our knowledge, no such reports are available in the literature for CrN or other similar nitride compounds in which RPES is used to differentiate the final electronic states of the specific feature though it is thoroughly used for strongly correlated TM oxide materials^49–51^.

#### X-ray absorption spectra

**Figure 3:**
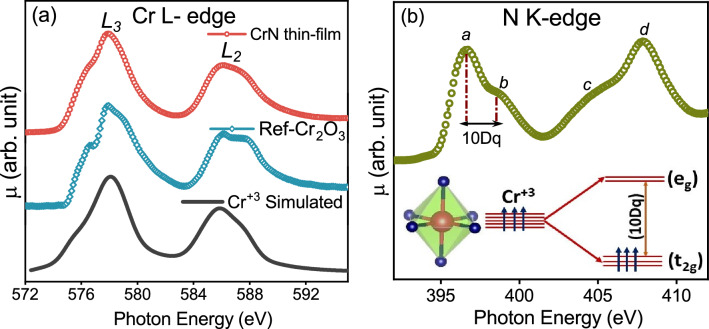
(**a**) X-ray absorption spectrum of Cr L-edges along with reference Cr$_2$O$_3$ and simulated Cr$^{+3}$ L-edge spectra (**b**) N K-edge X-ray absorption spectrum of the CrN thin film with a schematic shows the Cr-3$d$ orbital splitting into triply degenerated $\mathrm {t_{2g}}$ orbitals and doubly degenerated $\mathrm {e_g}$ orbitals due to ligand crystal field splitting. The XAS measurements have been carried out at 300 K.

Figure 3a presents Cr L$_{3,2}$ X-ray absorption spectra (XAS) of the CrN thin film along with the reference Cr$_2$O$_3$ bulk. The Cr L-edge spectra correspond to the transitions from a ground-state 2$p ^6$3$d ^3$ to one of the final-states 2$p ^5$3$d ^4$ allowed by the dipole selection rules ($\Delta$l = ± 1). The two broad peaks centered around $\approx$ 577.9 and $\approx$ 586 eV are assigned to L$_3$ (Cr: 2$p _{3/2}$-3$d$) and L$_2$ (Cr: 2$p _{1/2}$-3$d$) transitions, respectively, owing to the spin-orbit coupling. The relative position of the Cr L-edges and intensity ratio of $\mathrm {L_3:L_2}$ resemble with the Cr$_2$O$_3$ bulk reference as well as earlier reports^19,23^ confirming Cr$^{+3}$ valence state in the grown CrN thin film. The overall shape of the spin-orbit splitted Cr L$_{3,2}$ absorption edges is determined by the crystal field (CF) effects along with the multiplet effects, which are originated by 3$d$-3$d$ Coulomb interaction and the 2$p$-3$d$ Coulomb and exchange interactions^52^. Apart from the CF and multiplet states, the core-hole lifetime also contributes to the overall broadening of L$_{3,2}$ peaks^53^. Also, Cr L$_2$ peak is more broadened than L$_3$ due to the Coster–Kroning Auger decay process into the 2$p _{3/2}$ core-hole^52^. Further, the N K-edge XAS has been presented in Fig. 3b. Here, a sharp transition at a threshold of 396 eV can be seen. The absorption features *a* and *b* ascribed to the electronic transitions from N-1*s* core level to $\pi$ and $\sigma$ hybridized non-metal N-2*p* and metal Cr-3*d* t$_{2g}$ and e$_g$ orbitals, respectively while the feature *c* arises due to electronic transition into higher order hybridized Cr-4*s*4*p* and the N-2*p* orbitals. The crystal field energy (10Dq) is estimated to be $\approx$ 2 eV. The overall shape of Cr L$_3$ and L$_2$ edges is very sensitive to the 10Dq along with the Cr ground state (2$p ^6$3$d ^3$) as well as excited state (2$p ^5$3$d ^4$) multiplet, which can be controlled by the two-particle interaction parameter. Thus we have simulated the Cr L-edge spectra using a two-configuration charge transfer multiplet calculation for XAS and compared with the experimental spectrum (see Fig. 3a). The simulated Cr$^{+3}$ L-edge spectrum using $\Delta$ = 3.6 eV, U$_{2 p 3 d }$-U$_{3 d 3 d }$ = 1.9 eV, V(e$_g$) = 3 eV along with the reduction of slater integrals (F$^2$ and F$^4$) together with the appearance of pre-feature in the Cr L edge (see Fig. 3a) establishes the strong hybridization between Cr 3$d$-N 2$p$ orbitals. Thus, overall shape of Cr L$_{2,3}$ and N K edges spectra, value of 10Dq, Cr-2$p$ spin-orbit splitting derived from the $\mathrm {L_{3,2}}$ ($\approx$ 8.3 eV) are in good agreement with available reports on the stochiometric bulk confirming the stoichiometric nature of the grown CrN film with Cr-N hybridization strength and overall electronic structure resemble to the bulk^23^.

#### Experimental and first-principles electronic structure in the vicinity of Fermi-level

**Figure 4:**
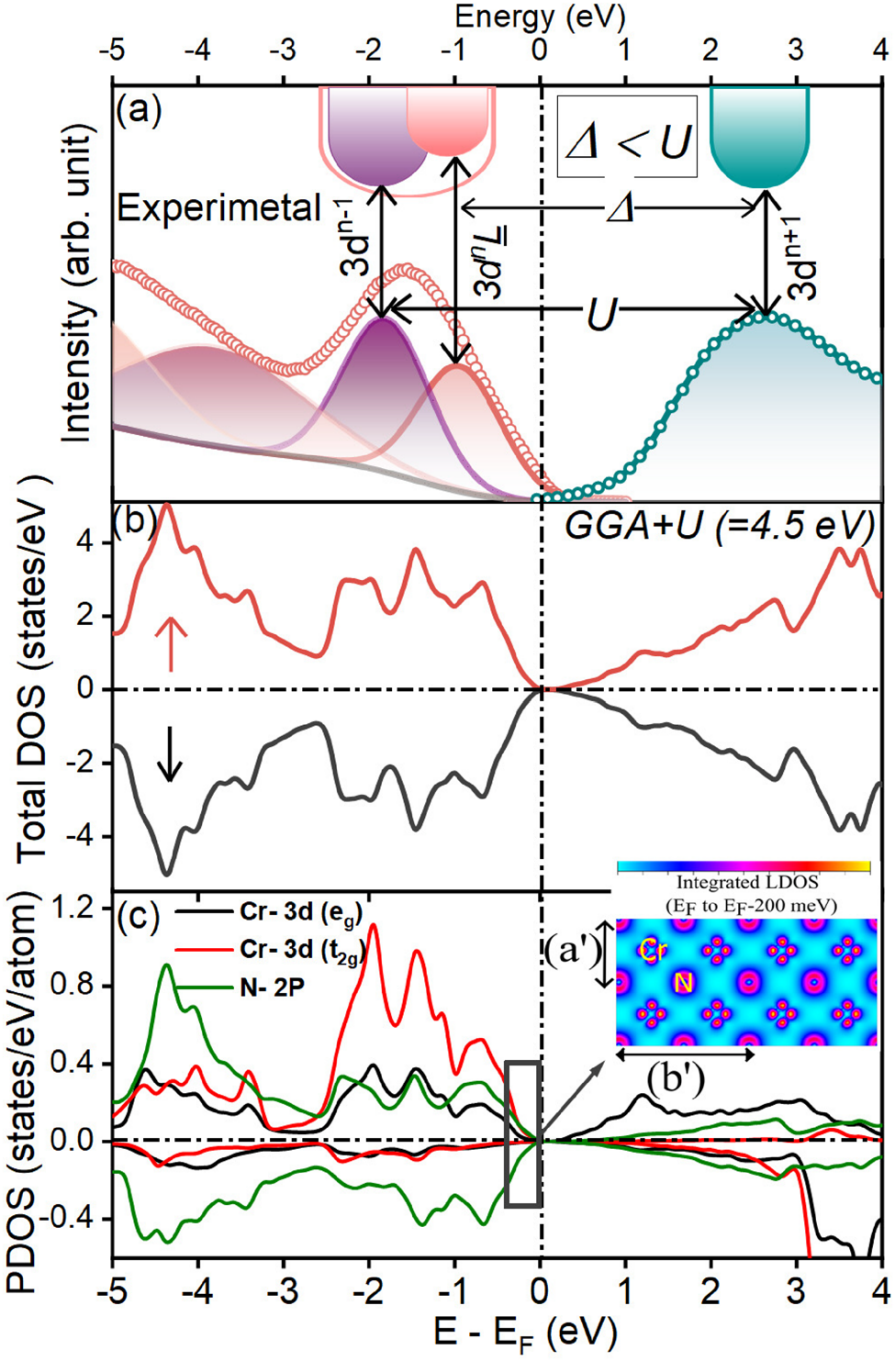
(**a**) Combined valence and conduction band of CrN thin film along with a schematic illustrating a charge-transfer-insulating nature of the grown CrN thin film. (**b**) Total electronic density of states (DOS) estimated using GGA + U scheme with U = 4.5 eV. (**c**) Orbital resolved partial density of states (PDOS) of CrN with a inset shows integrated local DOS plotted in the range of E$_F$ to E$_F$-200 meV in (001) viewing direction.

For a better understanding of the electronic structure near E$_F$, we have combined the experimental VBS and conduction band (CB) plotted in Fig. 4a. For the CB, N K-edge spectrum has been used, as it can be considered to represent the most weighted unoccupied character TM 3$d$ and TM 4$sp$ via the hybridization with ligand 2$p$ states. In addition, the photo induced core-hole effect on the final-state DOS is less severe compared to the TM 2$p$ edge^52^. To plot the CB, N K edge of CrN thin film was subtracted from the BE position of the rising tail of the N 2$p$ core-level photoelectron spectrum shown in Supplementary Fig. S2b of SM^54^. Although N K-edge XAS has been used for the CB mapping, it does not reflect the true DOS of the transition-metal states, rather reflecting the N 2$p$ projected metal 3$d$ DOS^55–57^. Hence, N K-edge XAS is used here as a CB (see Fig. 4a). The observed features in the band diagram shown in Fig. 4a are already been discussed in detail.

The nature of electronic state of grown CrN film is understood by the character of individual bands in the vicinity of E$_F$. These bands dictate the lowest energy charge fluctuations by evaluating the relative values of onsite Coulomb repulsion (U) and charge-transfer energy ($\Delta$). In the band structure, first band at 2.6 eV in CB is dominated by the Cr-3$d$ character, represents the spectroscopic signature of the upper Hubbard band (UHB) while features at 1.0 eV and 1.9 eV of VBS are assigned as Cr 3$d$-N 2$p$ hybridized and dominant Cr-3$d$ bands having 3$d ^n$ L and 3$d ^{n-1}$ final-state configurations, respectively (as already discussed in the RPES section). Hence, feature at 1.9 eV in VB is assigned to a lower Hubbard band (LHB). The onsite Coulomb repulsion energy ‘U’ (energy difference between the LHB and UHB) and charge-transfer energy ‘$\Delta$’ (energy difference between N-2$p$ band and UHB) are estimated as $\approx$ 4.5 eV and 3.6 eV, respectively. We examined the electronic structure of CrN using the first-principles calculations under GGA + U scheme and compared those to the experimental once (see Fig. 4a,b). In the GGA + U relaxed structure of CrN, a small distortion in atomic structure is seen (see Supplementary Fig. S3 of SM^54^). In the relaxed atomic structure of CrN, the local magnetic moment at Cr and N sites are $\approx$ 2.47 and 0.06 $\mu _B$, respectively that are within the range of experimental values^2^. The total DOS reveals salient features corroborate well with experimental results. Furthermore orbital resolved DOS indicates, along with Cr-3$d$ band, N-2$p$ band lies significantly near the E$_F$, signaling strong presence of N(2$p$)-Cr(3$d$) hybridized band character in the vicinity of E$_F$. It can be better visualized in the integrated local DOS shown in the inset of Fig. 4c plotted in the range of E$_F$ to E$_F$-200 meV dictates a solid contribution N-2$p$ band along with Cr-3$d$ bands. However, the dominance of Cr-3$d$ ($\approx$  thrice of N-2$p$) bands can seen in the range of − 1 to − 2.5 eV (see Fig. 4c). It validates our experimental findings that the dominant N-2$p$ band in the form of hybridized N(2$p$)-Cr(3$d$) is present near the E$_F$ while the Cr-3$d$ bands dominates away from E$_F$. Hence, the obtained experimental and first-principles results with $\Delta<$U suggest the lowest energy charge fluctuations between 3$d ^n$ L and 3$d ^{n+1}$ states occurs in CrN and thus, it is a charge-transfer-type insulator. As oxides of Cr are more ionic and thus have a less efficient screening than nitrides^26^. In a phenomenological model proposed by the Zaanen–Sawatzky–Allen, chromium oxide is suggested to be placed intermediate between Mott–Hubbard and charge-transfer regimes owing to the equivalent value of U $\approx$ $\Delta \approx$5 eV^58^. While the enhanced covalent nature of CrN results into the smaller value of $\Delta$, and thus lies in the charge transfer regime in contrast to oxide counterpart.

## Summary and conclusion

To summarize, we performed a comprehensive study to explore the electronic structure of CrN thin film using the complementary experimental techniques combined with the first-principles calculations. In-situ RHEED measurement confirms the relaxed and epitaxial nature of sputtered grown CrN (001) thin film on MgO (001) substrate. The electrical resistivity evidencing a clear first-order phase transition with a opening of small gap ($\approx$ 26 meV) in a high temperature regime. The overall spectral shape, absorption energy position of Cr L-edges confirms + 3 charge state and hopping parameters suggest strong hybridization between the N-2$p$ and Cr-3$d$ orbitals. The RPES study reveals a strong presence of N-2$p$ and Cr-3$d$ hybridized band near the E$_F$. Finally, experimental band structure combined with the theoretically estimated electronic DOS dictates the lowest energy charge fluctuations between 3$d ^n$ L and 3$d ^{n+1}$ states confirms the charge-transfer-type insulating ($\Delta<$U) state of the CrN thin film. Our results provide a better understanding of different competing electronic energetic that can be tailored using compressive/tensile strain as a results insulating or metallic states in the CrN can be stabilized^19,22^.

## Methods

### Experimental methods

CrN thin films were deposited on single-crystalline MgO (001) substrate using a reactive direct current magnetron sputtering system (AJA Int. Inc. Orion). The substrate temperature was fixed at 400 ${^{\circ }}$ C. The sputtering power was kept constant at 100 W during deposition. A mixture of N$_2$ (purity 99.999%) and Ar (purity 99.999%) gas was used to sputter Cr (purity 99.95%) target. The total gas flow during the sputtering process was kept constant at 50 standard cubic centimeter per minute (sccm) while the relative partial pressure of nitrogen defined as $\mathrm {R_{N_2} =P_{N_2}/(P_{Ar}+P_{N_2})}$; $\mathrm {P_{Ar}}$ and $\mathrm {P_{N_2}}$ are gas flow of Ar and N$_2$ gases, respectively was changed to deposit CrN thin films^59^. A base pressure of 4 $\times$ 10$^{-8}$ Torr was achieved in the vacuum chamber before deposition. The working pressure was 2.8 mTorr. In-situ reflection high energy electron diffraction (RHEED, KSA Instruments) with a Staib electron gun operating at an accelerating voltage of 35 keV, a beam current of 1.55 A and an emission current of 1 $\upmu$A was utilized to monitor the structural growth of CrN thin film on MgO (001) substrate. Ex-situ X-ray diffraction (XRD) measurements were performed using a standard diffractometer (Bruker D8 Advance) equipped with a Cu-K$_\alpha$ (1.54 Å) X-ray source. The temperature dependent four probe electrical resistivity measurements were carried out using a Quantum Design physical property measurement system. The X-ray near edge absorption spectroscopy (XANES) at Cr L$_{3,2}$ and N K-edges were carried out in the total electron yield (TEY) mode at soft X-ray beamline BL-01, Indus-2 at RRCAT, Indore, India. The energy resolution during XAS measurements across the measured energy range was $\approx$ 200 meV^60^. The pre and post-edge correction in the XANES were done using the Athena software^61^. The valence band spectrum measurements were performed at AIPES BL-02 beamline, Indus-1 synchrotron source at RRCAT, Indore, India. The vacuum in the experimental chamber during measurements was in the order of 10$^{-10}$ Torr. Prior to measurements the surface of thin film was cleaned using 500 eV Ar$^+$ ions at grazing incidence. The Au foil was kept in electrical contact with the sample holder for determination of the E$_F$. The experimental resolution was 300  meV in the measurement energy range.

### Theoretical methods

We simulated the Cr L-edge for Cr$^{+3}$ L-edge spectrum using the charge transfer multiplet program for x-ray absorption spectroscopy (CTM4XAS)^62^ under the ligand field and charge transfer multiplet approach. We performed charge transfer multiplet calculations by varying the reduction of Slater integrals, charge transfer energy ($\Delta$), d–d interaction energy, and N(2$p$)-Cr(3$d$) hybridization strength. For simulation, the Slater integrals were reduced to 80 % of the Hartree–Fock values and 10Dq in octahedral symmetry was set at 2 eV. The values of other parameters used for the simulation are as follows: charge transfer energy ($\Delta$) = 3.6 eV, U$_{2 p 3 d }$-U$_{3 d 3 d }$ = 1.9 eV, and hopping parameter V(e$_g$) = 3 eV. The Lorentzian and Gaussian line width of 0.25 eV and 0.3 eV, respectively are used for the simulation of spectrum, which accounts for L$_3$ core-hole lifetime and instrumental broadening respectively^52^.

Moreover, electronic structures are obtained from density functional theory (DFT) calculations by Quantum ESPRESSO code^63^. Norm-conserving pseudopotential with GGA-PBE functional for exchange and correlational energy was used. We have employed the Hubbard based DFT + U corrective scheme proposed by Andersen et al.^64^ and as implemented by Gironcoli et al.^65^ in our simulations. The onsite Hubbard parameter, U = 4.5 eV and Hund’s exchange term J$_H$=0 eV for Cr-3d state is used in this work which make U$_{eff}$ = U-J$_H$ = 4.5 eV. The kinetic energy cutoff for the plane wave was set at 680 eV. The orthogonal cell in its AFM [110]$_2$ configurations is constructed and a ball-and stick model is given in the SM^54^. The ground-state atomic structure was obtained by searching the low-energy atomic sites until forces on each atom were less than 10$^{-3}$ Ry/Bohr using the Broyden–Fletcher–Goldfarb–Shanno (BFGS) algorithm. The Brillouin Zone of AFM [110]$_2$ of CrN was sampled with a $\Gamma$-centered 6 $\times$ 12 $\times$ 8 mesh of k-points. The electronic eigenvalues were obtained over 24 $\times$ 48 $\times$ 32 k-mesh for densities of states analysis where a Gaussian broadening of 0.05 eV was used.

### Supplementary Information

Supplementary Information.

## Data Availability

All data generated or analysed during this study are included in the article that is available from the corresponding author.
