# Supplementary material for: Electronic correlations in epitaxial CrN thin film
Source: Sci Rep. 2023 Sep 25;13:15994. doi: 10.1038/s41598-023-42733-7 (PMC10519984; doi:10.1038/s41598-023-42733-7)
Supplement: Supplementary file 1 — Supplementary Information. [file 41598_2023_42733_MOESM1_ESM.pdf]

Supplemental material “Electronic Correlations in Epitaxial CrN Thin film”

Shailesh Kalal[1], Sanjay Nayak[2], Sophia Sahoo[1], Rajeev Joshi[1], Ram Janay Choudhary[1], Rajeev Rawat[1], and Mukul Gupta[1] *

[1]*UGC-DAE Consortium for Scientific Research,* University Campus, Khandwa Road, Indore 452 001, India and

[2] Thin Film Physics Division, Department of Physics, Chemistry and Biology (IFM), Linköping University, SE-581 83, Linköping, Sweden

Email: mgupta@csr.res.in

$k_\perp$ and photon energy plot as a function of binding energy:

We have presented binding energy and photon energy profiles as functions of $k_\perp$ in Fig. S1. The determination of $k_\perp$ is facilitated through the utilization of the following relation:

$$k_\perp = \sqrt{\frac{-2m}{h}[E_{K.E.} cos^2\theta + V_0]}$$

Here, $E_{K.E.}$ represents the kinetic energy of photoelectrons, θ denotes the emission angle and V_0 $(10\ eV)$ stands for the inner potential of CrN. The 2nd bulk gamma point in $k_\perp$ direction (Γ_2) lies at $k_\perp = 2\pi/c = 1.51$ Å^{-1} that cannot be reached within the measured photon energy range while the X point lies at 2.3 Å^{-1}. It is important to note that our analysis reveals a slightly dispersed nature of the A, F and C bands, which is an indication of the 3d nature of bands while no clear dispersion is visible in other bands which may be due to the 2d or quasi 2d nature of band or limiting instrumental resolution (≈ 300 meV).

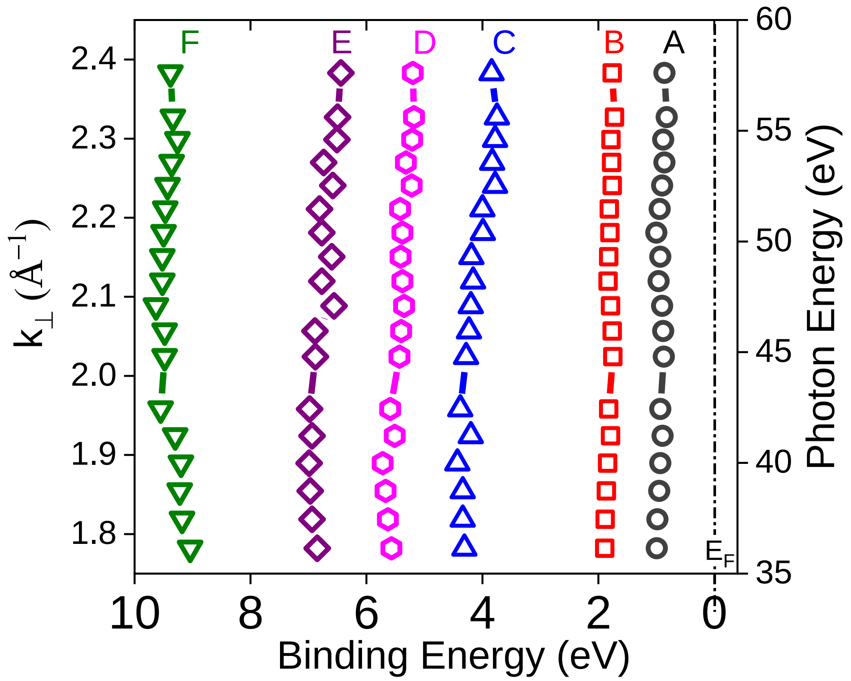

Figure S1. $k_\perp$ and photon energy as a function of binding energy for features A, B, C, D, E and F.

X-ray photoelectron spectroscopy measurements-

The nitrogen stoichiometry in CrN is known to influence its properties. To check the correct chemical states and stoichiometry along with XAS, Cr-2P and N-1s core level has been probed using the x-ray photoemission spectroscopy (XPS) measurements. The XPS measurement has been performed on the CrN thin film deposited at R_{N2}=30% using a Al K_{α} (1486.6 eV) x-ray source with the instrumental resolution ≈ 0.8 eV. Fig. S2 (a) and (b) show the Cr-2*p* and N1*s* core-level spectrum, respectively. Here, the low binding energy (BE) metallic screening feature (pre-edge feature) is not resolved [1]. The fitted XPS spectra have been shown in Fig. S2. From the fitted XPS data we observed the well-screened features (A, C) at the lower BE side of the main spin-orbit splitted (Cr-$2p_{3/2}$ & $2p_{1/2}$) features (B, D). These spectra are deconvoluted using the combination of the Gaussian and Lorentzian and the binding energy position agrees with the earlier reports again confirming the +3 charge state of Cr. The N-1s spectrum is well-fitted using a single peak confirming the correct chemical order [1,2].

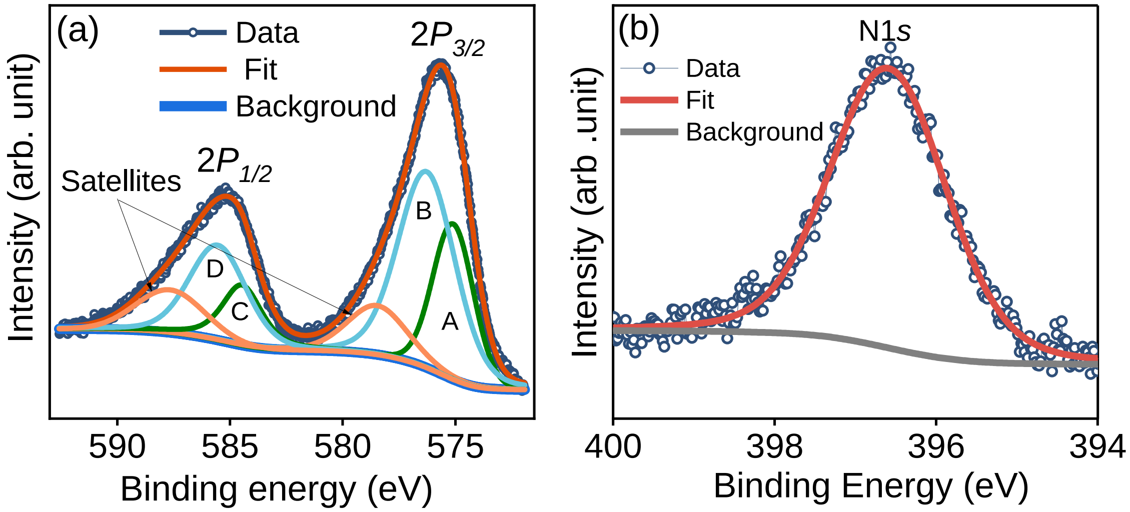

Figure S2. (a) Cr 2*p* and (b) N 1*s* core level x-ray photoemission spectrum of the CrN thin film deposited at R_{N2} = 30%.

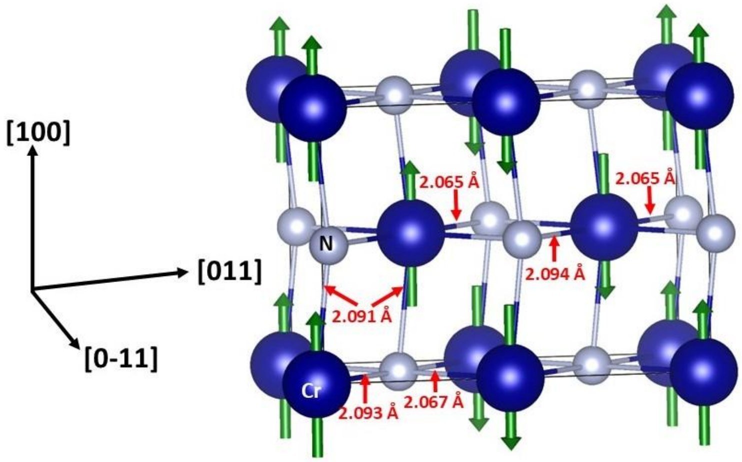

Figure S3. DFT estimated the orthorhombic cell of CrN with AFM $[110]_2$ spin ordering.

References:

[1]. P. Bhobe, A. Chainani, M. Taguchi, T. Takeuchi, R. Eguchi, M. Matsunami, K. Ishizaka, Y. Takata, M. Oura, Y. Senba, H. Ohashi, Y. Nishino, M. Yabashi, K. Tamasaku, T. Ishikawa, K. Takenaka, H. Takagi, and S. Shin, Evidence for a correlated insulator to antiferromagnetic metal transition in CrN, Physical review letters **104**, 236404 (2010).

[2]. Z. Hui, X. Tang, R. Wei, L. Hu, J. Yang, H. Luo, J. Dai, W. Song, X. Liu, X. Zhu, Y. Sun, Facile chemical solution deposition of nanocrystalline CrN thin films with low magnetoresistance, RSC Advances. 4 (2014) 12568–12571.
